# Amides from *Piper capense* with CNS Activity – A Preliminary SAR Analysis

**DOI:** 10.3390/molecules14093833

**Published:** 2009-09-25

**Authors:** Mikael E. Pedersen, Bjørn Metzler, Gary I. Stafford, Johannes van Staden, Anna K. Jäger, Hasse B. Rasmussen

**Affiliations:** 1 Department of Medicinal Chemistry, Faculty of Pharmaceutical Sciences, University of Copenhagen, Universitetsparken 2, DK-2100 Copenhagen, Denmark; E-Mails: mep@farma.ku.dk (M-E.P.); b.metzler@yahoo.dk (B.M.); ankj@farma.ku.dk (A-K.J.); 2 Research Centre for Plant Growth and Development, School of Biological and Conservation Sciences, University of KwaZulu-Natal, Pietermaritzburg, Private Bag X01, Scottsville 3209, South Africa; E-Mails: garyistafford@gmail.com (G-I.S.); Vanstaden@ukzn.ca.za (J.V-S.)

**Keywords:** *Piper capense*, piperine, GABA, benzodiazepine, epilepsy, multi target effect

## Abstract

*Piper capense* L.f. (Piperaceae) is used traditionally in South Africa as a sleep inducing remedy. Bioassay-guided fractionation of the roots of *P. capense* led to the isolation of piperine (**1**) and 4,5-dihydropiperine (**2**), which showed moderate affinity for the benzodiazepine site on the GABA_A_ receptor (IC_50_ values of 1.2 mM and 1.0 mM, respectively). The present study suggests that strict structural properties of the amides are essential for affinity. Taken together, these observations suggest that the carbon chain must contain not less than four carbons, and that a conjugated double bond, adjacent to the amide group, is necessary for binding to the receptor and that the amine part should be bulky.

## Introduction

Epilepsy is a chronic disorder caused by a sudden imbalance between the inhibitory and excitatory signals in the brain with γ-aminobutyric acid (GABA) and glutamate, respectively. It is characterized by recurrent seizures, leading to a variety of medical and psychosocial problems. For many years, treatment of epilepsy has focused on the GABAergic or the glutamatergic system, though other important neurotransmitter systems have been investigated, including the noradrenaline and voltage-gated sodium channels. Benzodiazepines are an important group of antiepileptic compounds with anxiolytic, anticonvulsant, muscle relaxant and sedative-hypnotic effects. However, clinical treatment of epilepsy, which consists of keeping unprovoked and recurrent seizures under control, still warrant the development of new alternative drugs, as the existing treatments fail to control all types of epilepsy [1,2,3,4]. 

The tuber or roots of *Piper capense* L.f. (Piperaceae) are used traditionally in South Africa as a sleep inducing remedy [5]. In a screening of plants used for sedation or for treating CNS related ailments, the ethanol extract of the roots showed affinity for the benzodiazepine site on the GABA_A_ receptor [6], thus indicating the presence of potential antiepileptic compounds. 

## Results and Discussion

**Figure 1:**
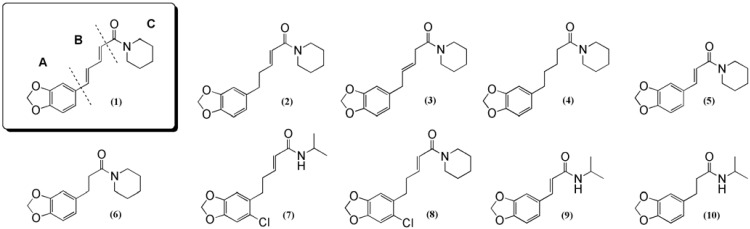
Compounds tested.

In the present study, bioassay-guided fractionation led to the isolation of the two amides, namely piperine (**1**) and 4,5-dihydropiperine (**2**) (Figure 1), which showed low affinity for the benzodiazepine binding site on the GABA_A_ receptor with K_i_ values of 1.175 ± 0.003 mM and 1.054 ± 0.004 mM, respectively (Table 1). The NMR and MS data were identical with previously published data for (**1**) and (**2**) [7,8].

To obtain sufficient amount of dihydropiperine (**2**) for testing and to investigate the structure-activity requirements of these amides, nine compounds were synthesized in which the B and C moieties of piperine were modified, resulting in a small library of ten amides for pharmacological testing (Figure 1). 

None of the synthesized amides improved the affinity for the benzodiazepine binding site, however, indicating that strict structural requirements have to be met in order for the compounds to bind. Compound **8** showed affinity comparable with that of the corresponding non-chlorinated analog **2**, (**2**, IC_50_ = 1.054 ± 0.004 mM versus **8**, IC_50_ = 1.782 ± 0.003). 

**Table 1:** Binding results.

Compounds	[^3^H]-flumazenil binding assay IC_50_ (mM)	[^3^H]-citalopram binding assay IC_50_ (mM)
1	1.175 ± 0.003	n.d.
2	1.054 ± 0.004	2.979 ± 1.627
3	n.d.	n.d.
4	n.d.	0.585 ± 1.520
5	n.d.	n.d.
6	n.d.	n.d.
7	n.d.	n.d.
8	n.d.	2.692 ± 1.387
9	n.d.	n.d.
10	n.d.	n.d.

Data are presented as mean ± standard error. n.d.- not detected in tested concentrations below 10 mM.

Apparently, the linker (B) must no be shorter than four carbons and should contain a minimum of one double bond, adjacent to the amide group, since moving the double bond to the β,γ-position proved detrimental for binding to the receptor. It appears, also, that the C moiety (the amine part) must be bulky as no affinity was found for the corresponding isopropyl amides.

Numerous pharmacological studies have been conducted on piperine and its derivatives, and its sedative and antiepileptic properties have been demonstrated [9,10,11]. Antiepilepsirin (**5**), a cinnamamide also isolated from *P. capense* during the present study, has shown promising results and has entered clinical trials in China [11]. Interestingly, antiepilepsirin showed no affinity for the benzodiazepine site, in the present study, when tested in the [^3^H]-flumazenil binding assay, suggesting that it exerts its effect through a different mode of action.

A structure-activity study, using more than 200 synthesized cinnamamides on MES seizures in mice tried to map the structure-activity [12]. The optimal molecular design included high lipophilicity (LogP = 3.8-4.3) and small, hydrophobic and electron-withdrawing substituents on the aromatic ring. The aromatic ring and the amide group on the double bond should have an *(E)-*configuration and be separated by at least two carbon atoms [12]. The dihydro-cinnamamides used in the present study (including **6** and **10**) have shown protection against MES induced seizures [12], despite the lack of affinity for the benzodiazepine site on the GABA_A_ receptor, indicating a different mode of action. 

Studies on the extract of *Piper methysticum* Forster f. (Piperaceae) have demonstrated the GABA agonist activity of the kavalactones [13,14]. Methysticin, a kavalactone from *P. methysticum* shares some structural similarities with the isolated amides from *P. capense*. The activation of the GABA_A_ receptor by methysticin, however, was shown not to be associated with the benzodiazepine binding site [15].

The testing of the compounds **1-10** in the serotonin re-uptake assay showed that compounds **2**, **4** and **8** had low affinity for the serotonin re-uptake transport protein with K_i_ values of 2.979 ± 1.626 mM; 0.585 ± 1.520 mM and 2.692 ± 1.387 mM, respectively. This could indicate that the sedative properties of *Piper capense* could be a result of a mixture of closely related compounds acting jointly on the serotonergic and GABAergic system.

## Conclusions

The bioassay-guided fractionation of the roots of *P. capense* resulted in the isolation of two amides **1** and **2** with low affinity for the benzodiazepine site on the GABA_A_ receptor. Structure-activity studies did not reveal compounds with higher affinity, indicating that strict structural requirements have to be met for receptor binding. In view of the moderate binding affinity of piperine (**1**) and dihydropiperine (**2**) for the GABA_A_ receptor and the fact that cinnamamides and kavapyrones showing anticonvulsant activity are also found in *Piper* species, albeit working through a different mode of action, it is likely that the reason for the pronounced antiepileptic activity of *Piper* extracts is due to a multi target effect. Different compounds acting on different targets with moderate activity, either additively or synergistically, will give a response, which is larger than that shown by the individual compounds. This is an effect found frequently for natural products extracts and remedies [16,17].

## Experimental

### General

NMR spectra were obtained at 25 °C on a Varian Mercury 300 MHz spectrometer in chloroform-*d*, using TMS as internal standard (^1^H-NMR at 300 MHz, ^13^C-NMR at 75 MHz). GC-MS (Agilent 6890N GC system; 7683 series injector; 5973N Mass Selective Detector) was used to determine purity and corroborate the structure of the amides. HR-MS data were obtained on a Micromass Q-TOF spectrometer in the positive mode using electrospray ionization (ESI). HPLC system I consisted of a semi-preparative column (Phenomenex® Luna C-18 column, 5 μm, 250 mm x 21 mm with a guard column Phenomenex® Luna C-18, 5 μm, 50 mm x 21 mm) and was employed using Dionex P580 pumps, a Dionex PDA-100 Photodiode Array UV–vis Detector, a Dionex ASI 100 Auto Sample Injector and a Foxy Junior fraction collector using a gradient of MeCN and water (60–100% MeCN at *t* = 0–60 min, 100% MeCN at t = 60–70 min and 100–60% MeCN at *t* = 70–85 min). The flow rate was 7 ml/min and detection was done at 200–600 nm. HPLC system II consisted of an analytic column (Luna® C-18, 5 μm, 150 mm × 4.6 mm) with the isocratic system of 35% MeCN in water. The flow rate was 1 mL/min and detection was done at 200–600 nm. 

### Plant material

Roots of *Piper capense* L.f. (Piperaceae) were collected near Pietermaritzburg, KwaZulu-Natal, South Africa and a voucher specimen (Stafford 89 NU) has been deposited in the Bews’ Herbarium at the University of KwaZulu-Natal, Pietermaritzburg.

### Bioassay

The GABA binding assay was carried out according to a previously published method [18], with modifications [20,21]. To a suspension of rat brain cortex (500 µL) was added test solution (25 µL) and [^3^H]-flumazenil (25 µL, Ro 15-1788, Perkin-Elmer Life Science) to 0.5 nM final concentration in the assay. The suspension was mixed and incubated for 40 min on ice and poured directly onto glass fiber filters (Adventic, GC-50) under suction, in an ice-bath, followed by washing with ice-cold Tris-citrate buffer. The amount of radioactivity was determined by liquid scintillation counting. Non-specific binding was determined using diazepam (1 µM final concentration in the assay) and flumazenil was used as positive control. Specific binding was calculated as total binding minus non-specific binding. All experiments were done in triplicate. The serotonin re-uptake inhibition assay was performed according to previously described methods [22].

### Extraction and isolation

Ground dry plant material (100 g) was extracted three times with ethanol (1:10 w/V) for 60 min with sonication. The extract was filtered and evaporated to dryness under reduced pressure. The residue (1.97 g) was dissolved in EtOAc (10 mL) and partitioned three times against water (100 mL) to remove inactive polar compounds. The combined organic phases were evaporated and the residue (0.885 g) fractionated on a VLC column (7x10 cm i.d.) with solvent mixtures of increasing polarity, to afford six fractions. Fraction 1 (165 mg) was eluted with toluene (200 mL); fraction 2 (585 mg) was eluted with toluene/EtOAc (80:20, 200 mL); fraction 3 was eluted with toluene/EtOAc (50:50, 200 mL); fraction 4 (47 mg) was eluted with eluted with toluene/EtOAc (20:80, 200 mL); fraction 5 was eluted with EtOAc (200 mL) and fraction 6 (21 mg) was eluted with MeOH (400 mL). Fraction 2, which was the most active in the bioassay, was submitted to reversed-phase HPLC (HPLC-system I). This fractionation afforded five fractions. Further HPLC fractionation (HPLC-system II) of the active fraction 3 (32.2 mg) on an analytic column led to the isolation and identification of **1** (5.3 mg) and **2** (2.6 mg) (Figure 1) as the active components.

*Piperine* (**1**): ^1^H-NMR δ (ppm): 7.40 (ddd, J = 15.0 Hz, *J* = 1.6 Hz, *J* = 8.0 Hz, 1H), 6.98 (d, *J* = 1.6 Hz, 1H), 6.89 (dd, *J* = 8.0 Hz, *J* = 1.6 Hz, 1H), 6.78 (d, *J* = 8.0 Hz, 1H), 6.77 (d, *J* = 15.0 Hz, 1H), 6.76 (dd, *J* = 8.5 Hz, *J* = 15 Hz, 1H), 6.44 (d, *J* = 15.0 Hz, 1H), 5.98 (s, 2H); 3.63 (br s, 2H), 3.52 (br s, 2H), 1.55-1.70 (m, 6H; MS: *m/z* 285.14 [M]^+^.

*4,5-Dihydropiperine* (**2**): ^1^H-NMR δ (ppm): 6.70 (dt, *J* = 15.1 Hz, *J* = 7.0 Hz, 1H), 6.65 (d, *J* = 7.8 Hz, 1H), 6.60 (d, *J* = 1.7 Hz, 1H), 6.50 (dd, *J* = 7.8 Hz, *J* = 1.7 Hz, 1H), 6.18 (dt, *J* = 15.0 Hz, *J* = 1.5 Hz, 1H), 5.80 (s, 2H), 3.55 (br s, 2H), 3.35 (br s, 2H), 2.60 (t, *J* = 7.6 Hz, 2H), 2.40 (dt, *J* = 7.3 Hz, J = 1.3 Hz, 2H), 1.55 (m, 6H); MS: m*/z* 287.15 [M]^+^.

### Synthesis of piperine analogs

**Scheme 1:**
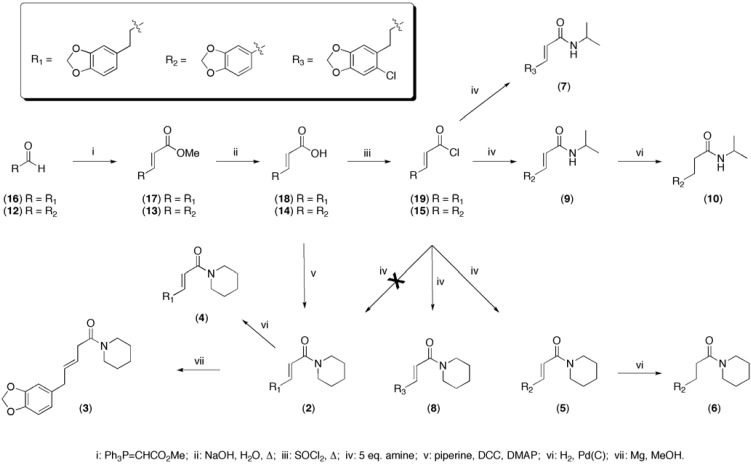
Synthesis of compounds **2**-**10**.

To assess the structure-activity relationships a small library of ten synthesized analogs was tested in the bioassay (Scheme 1). Wittig reaction between commercially available piperonal (**12**) and methyl triphenylphosphoranylidene acetate afforded the α,β-unsaturated ester **13** in high yield. The methyl ester was hydrolysed with 1 M NaOH and converted to the corresponding acid chloride **15** using thionyl chloride. The two amides **5 + 10** (Figure 1) were synthesized from **15**. The last three steps from ester to amide were performed in one pot, without purification. In order to obtain sufficient material for rigorous testing, a synthesis of **2** was performed using same method as described above (Scheme 1), starting with the homologous aldehyde **16** as starting material. Unexpectedly, the last step yielded the chlorinated analog **8**. The chlorinated isopropyl analog **7** was likewise synthesized using the method described above. Dihydropiperine **2** was subsequently synthesized through a DCC coupling of the acid with piperidine. The last four analogs were synthesized directly from other synthesized test compounds by reduction of the double bonds under standard (H_2_/Pd) conditions (Figure 2) and the transposed dihydropiperine **3** was synthesized by reduction of piperine with magnesium in methanol [23]. 

*trans-3-Benzo[1,3]dioxol-5-yl-acrylic acid methyl ester* (**13**): Piperonal (**12**; 1.5 g, 10 mmol) was dissolved in DCM (40 mL) and the mixture cooled in an ice-water bath to 5 ºC. Methyl triphenylphosphoranylidene acetate (5.04 g, 15 mmol), dissolved in DCM (50 mL), was added drop- wise and the mixture stirred for 24 hours at room temperature and evaporated under reduced pressure. The crude product was purified by flash-silica gel chromatography (EtOAc/heptane 1:1) to afford the title compound as colorless oil. (1.9 g; 86%; TLC *R_f_* 0.50, EtOAc/heptane, 1:2). ^1^H-NMR δ (ppm): 7.56-7.62 (d, 1H), 7.02 (m, 1H), 6.98 (d, 1H), 6.79-6.82 (d, 1H), 6.25-6.30 (d, 1H), 6.00 (s, 2H), 3.79 (s, 3H).

*trans-3-Benzo[1,3]dioxol-5-yl-acrylic acid* (**14**): 3-Benzo[1,3]dioxol-5-yl-acrylic acid methyl ester (**13**; 1.9 g, 9.2 mmol) was dissolved in THF (50 mL). To the mixture was added 1M NaOH (50 mL) and the mixture stirred for 24 hours. THF was removed under reduced pressure and conc. HCl added (pH < 3). The slurry was filtered and the title compound obtained as white crystals, which were dried at reduced pressure and used directly in the following synthesis. Yield: (1.76 g, 99%).

*trans-3-Benzo[1,3]dioxol-5-yl-acryloyl chloride* (**15**): 3-Benzo[1,3]dioxol-5-yl-acrylic acid (**14**; 6.0 g, 31.2 mmol) was dissolved in thionyl chloride (3.7 g, 312 mmol). The reaction was heated at reflux for 1 hour and concentrated under reduced pressure to afford the title compound. The compound was used directly in the following reaction.

*trans-3-Benzo[1,3]dioxol-5-yl-N-isopropyl-acrylamide* (**9**): 3-Benzo[1,3]dioxol-5-yl-acryloyl chloride (**15**; 0.5 g; 2.38 mmol) was added to a solution of isopropylamine (0.85 g; 14 mmol) in DCM (20 mL). The reaction was stirred at room temperature for 1 hour and concentrated under reduced pressure. The crude product was dissolved in EtOAc (50 mL) and the organic phase washed with 4 M HCl and 1M NaOH. To the organic phase was added heptane (50 mL) and the mixture concentrated slowly, at reduced pressure, until crystals were seen. The reaction was cooled and the product collected by filtration leaving the title compound as white crystals. (305 mg, 55%; TLC *R_f_* 0.6, EtOAc/heptane, 1:2). ^1^H-NMR δ (ppm): 7.53-7.48 (d, 1H), 6.94 (d+s, 2H), 6.76 (d, 1H), 6.22 (d, 1H), 5.97 (s, 2H), 5.55 (bs, 1H), 4.21 (m 1H), 1.21 (d. 6H); ^13^C-NMR: δ (ppm): 165.88, 148.40, 148.39, 140.66, 129.52, 123.99, 119.28, 108.73, 106.50, 101.63, 41.77, 23.09; MS: *m/z* 233.11 [M]^+^; HRMS (ESI+), M+Na^+^: 256.0944; calculated for C_13_H_15_NO_3_Na: 256.0950. 

*Antiepilepsirine* (**5**): 3-Benzo[1,3]dioxol-5-yl-acryloyl chloride (**15**; 0.5 g; 2.38 mmol) was added to a solution of piperidine (0.85 g; 10 mmol) in DCM (20 mL). The reaction was stirred at rt. for 1 hour and concentrated under reduced pressure. The crude product was dissolved in 50 mL EtOAc and the organic phase washed with 4 M HCl and then 1M NaOH. The product was obtained as described for **(9)** leaving the title compound as white crystals. (85 mg; 14%; TLC *R_f_* 0.63, EtOAc/heptane, 1:2).). ^1^H-NMR δ (ppm): (see above); ^13^C-NMR δ (ppm): 167.01, 149.03, 148.39, 142.18, 130.18, 123.83, 115.86, 108.70, 106.54, 101.62, 48.03, 44.01, 26.00, 25.50, 24.89; MS: *m/z* 259.12 [M]^+^; HRMS (ESI+), M+H^+^: 260.1295; calculated for C_15_H_18_NO_3_: 260.1287.

*trans-5-Benzo[1,3]dioxol-5-yl-pent-2-enoic acid methyl ester* (**17**): 3-Benzo[1,3]dioxol-5-yl-propionaldehyde (**16**; 0.48 g, 2.70 mmol) was dissolved in DCM (10 mL). The reaction mixture was cooled on an ice-water bath to 5 ºC. Methyl triphenylphosphoranylidene acetate (1.35 g, 4 mmol), dissolved in DCM (20 mL), was added drop wise to the reaction. The mixture was stirred for 24 hours at room temperature and evaporated under reduced pressure. The crude product was purified by flash chromatography (EtOAc/heptane 1:1) to afford the title compound as oil. (410 mg, 65%; TLC *R_f_* 0.63, EtOAc/heptane, 1:2) ^1^H-NMR: δ 6.91 (m, 1H), 6.68 (d, 1H), 6.60 (d, 1H), 6.50 (s, 1H), 6.95 (s, 2H), 5.82 (d, 1H), 3.67 (s, 3H), 2.67 (t, 2H), 2.45 (m, 2H); MS: *m/z* 234.09 [M]^+^.

*trans-5-Benzo[1,3]dioxol-5-yl-pent-2-enoic acid* (**18**): 5-Benzo[1,3]dioxol-5-yl-pent-2-enoic acid methyl ester (**20**; 0.61 mg, 2.6 mmol) was dissolved in THF (10 mL). To the reaction mixture was added 1M NaOH (10 mL) and the mixture stirred for 72 hours. THF was removed under reduced pressure and conc. HCl was added (pH < 3). The water phase was washed with EtOAc and the organic phases combined and concentrated under reduced pressure. The crude product was purified by flash chromatography (EtOAc/heptane 1:1) to afford the title compound as white crystals, which were dried under reduced pressure. (450 mg, 78%; TLC *R_f_* 0.33, EtOAc/heptane, 1:2); ^1^H-NMR δ (ppm): 7.05 (m, 1H), 6.8-6.7 (m, 3H), 5.92 (s, 2H), 5.80 (d, 1H), 2.68 (m, 2H), 2.46 (m, 2H). 

*trans-5-Benzo[1,3]dioxol-5-yl-pent-2-enoyl chloride* (**19**): 5-Benzo[1,3]dioxol-5-yl-pent-2-enoic acid (**18**; 385 mg, 1.75 mmol) was dissolved in thionyl chloride (1.2 g, 10.4 mmol). The reaction was heated to reflux for 1 hour and, then concentrated under reduced pressure to afford the title compound quantitatively, which was used directly in the following reaction. 

*trans-5-(6-Chlorobenzo[1,3]dioxol-5-yl)-N-isopropylpent-2-enamide* (**7**): 5-Benzo[1,3]dioxol-5-yl-pent- 2-enoyl chloride (**19**; 208 mg; 0.88 mmol) was added to a solution of isopropylamine (0.85 g; 14 mmol) in DCM (10 mL). The reaction was stirred at room temperature for 1 hour and concentrated under reduced pressure. The crude product was dissolved in EtOAc (20 mL) and the organic phase washed with 4 M HCl and 1M NaOH. The product was obtained as described for **(10)** and further purified using HPLC-system II leaving the product as white crystals. ^1^H-NMR δ (ppm): 6.75 (s, 1H), 6.60 (s, 1H), 6.60 (d, *J* = 1.7 Hz, 1H), 6.18 (dt, *J* = 15.0 Hz, *J* = 1.5 Hz, 1H), 5.85 (s, 2H), 4.10 (m, 1H), 2.60 (t, *J* = 7.3 Hz, 2H), 2.40 (dt, *J* = 7.3 Hz, *J* = 1.3 Hz, 2H), 1.20 (s, 6H); MS: *m/z* 295.10 [M]^+^; HRMS (ESI+), M+H^+^: 296.1052; calculated for C_15_H_19_NO_3_Cl: 296.1053.

*trans-5-(6-Chlorobenzo[1,3]dioxol-5-yl)-1-(piperidin-1-yl)pent-2-en-1-one* (**8**): 5-Benzo[1,3]di-oxol-5-yl-pent-2-enoyl chloride (**19**; 208 mg; 0.88 mmol) was added to a solution of piperidine (0.85 g; 10 mmol) in DCM (10 mL). The reaction was stirred at room temperature for 1 hour and concentrated under reduced pressure. The crude product was dissolved in EtOAc (20 mL) and the organic phase washed with 4M HCl and then 1M NaOH. The product was obtained as described for **(10)** and further purified using HPLC-system II leaving the product as white crystals. ^1^H-NMR δ (ppm): 6.75 (s, 1H), 6.60 (s, 1H), 5.85 (s, 2H), 3.56 (br s, 2H), 3.35 (br s, 2H), 2.60 (t, *J* = 7.6 Hz, 2H), 2.40 (dt, *J* = 7.3 Hz, *J* = 1.3 Hz, 2H), 1.56 (m, 6H); MS: *m/z* 321.11 [M]^+^; HRMS (ESI+), M+Na^+^: 344.1034; calculated for C_17_H_20_NO_3_NaCl: 344.1029.

*4,5-Dihydropiperine* (**2**): A mixture of the carboxylic acid (**21;** 440 mg; 1 mol), piperidine (1.9 g; 22 mol), dicyclohexylcarbodiimide (DCC, 412 mg; 2 mmol), 4-(dimethylamino)pyridine (DMAP, 244 mg; 2 mmol) in dichloromethane (10 mL) was stirred at room temperature for 2 h. The clear solution became turbid after 5 minutes. The precipitated dicyclohexylurea was filtered off and washed with dichloromethane. The combined organic phases were extracted with a saturated aqueous citric acid solution and water, dried (MgSO_4_) and evaporated to give the corresponding amide which was purified by flash chromatography (EtOAc/heptane 1:1) to afford the title compound, which was re-crystallized (EtOAc/heptane) to afford **2** as white crystals. (80 mg; 14%; TLC *R_f_* 0.58, EtOAc/heptane, 1:2) ^1^H-NMR (CDCl_3_) (data: see above).

*(E)-5-(Benzo[1,3]dioxol-5-yl)-1-(piperidin-1-yl)pent-3-en-1-one* (**3**): To piperine **1** (1 g, 3.5 mmol) in methanol (20 mL) was added magnesium powder (91 mg, 3.5 mmol) and the mixture stirred at room temperature for 2 hrs. The reaction mixture was filtered and the methanol removed under reduced pressure. The residue was neutralized with a 1 M NH_4_Cl solution (25 mL) and extracted with chloroform. The extract was concentrated to a gummy mass, which was purified by VLC over silica gel using hexane/EtOAc (1:1) as eluant to afford **3** as a syrup. (0.95 g, 95%; TLC *R_f_* 0.55, EtOAc/heptane, 1:2). ^1^H-NMR δ (ppm): 6.75 (s, 1H), 6.60 (s, 1H), 6.60 (d, *J* = 1.7 Hz, 1H), 6.18 (dt, *J* = 15.0 Hz, *J* = 1.5 Hz, 1H), 5.85 (s, 2H), 5.60 (m, 4H), 3.5 (m, 4H), 3.3 (d, 2H), 3.1 (d, 2H), 1.56 (m, 6H); MS: *m/z* 287.15 [M]^+^; HRMS (ESI+), M+H^+^: 288.1600; calculated for C_17_H_22_NO_3_: 288.1600.

*Tetrahydropiperidine* (**4**): Piperine (**1,** 1g, 3.5 mmol) was dissolved in MeOH (20 mL) and Pd(C) was added (50 mg). The flask was closed and put under a H_2_ atmosphere for 6 hours. The reaction mixture was filtered and the organic phase concentrated under reduced pressure to afford the title compound as orange oil, which was purified by VLC over silica gel using hexane/EtOAc (1:1) as eluant to afford **3**. (0.95 g, 95%; TLC *R_f_* 0.60, EtOAc/heptane, 1:2). ^1^H-NMR δ (ppm): 6.75 (s, 1H), 6.60 (s, 1H), 6.60 (d, *J* = 1.7 Hz, 1H), 6.18 (dt, *J* = 15.0 Hz, *J* = 1.5 Hz, 1H), 5.85 (s, 2H), 3.5 (m, 4H), 2.6 (t, 2H), 2.4 (t, 3H), 1.6 (m, 12H); MS: *m/z* 289.17 [M]^+^; HRMS (ESI+), M+H^+^: 290.1748; calculated for C_17_H_24_NO_3_: 290.1756.

*3-Benzo[1,3]dioxol-5-yl-1-piperidin-1-yl-propan-1-one* (**6**): The amide **5** (1 g, 3.86 mmol) was dissolved in MeOH (20 mL), Pd(C) (50 mg) was added and the flask closed and put under a H_2_ atmosphere for 4 hours. The reaction mixture was filtered and the organic phase concentrated under reduced pressure to afford the title compound as brown crystals, which were purified using HPLC-system II. (0.99 g, 95%; TLC *R_f_* 0.50, EtOAc/heptane, 1:2). ^1^H-NMR δ (ppm): 6.75 (s, 1H), 6.60 (s, 1H), 6.60 (d, *J* = 1.7 Hz, 1H), 6.18 (dt, *J* = 15.0 Hz, *J* = 1.5 Hz, 1H), 5.85 (s, 2H), 3.5 (m, 4H), 2.6 (t, 2H), 2.4 (t, 3H), 1.6 (m, 12H); MS: *m/z* 261.14 [M]^+^; HRMS (ESI+), M+Na^+^: 284.1273; calculated for C_15_H_19_NO_3_Na: 284.1263.

*3-Benzo[1,3]dioxol-5-yl-N-isopropyl-propionamide* (**10**): Compound **9** (1g, 4.02 mmol) was dissolved in MeOH (20 mL) and Pd(C) was added (50 mg). The flask was closed and put under a H_2_ atmosphere for 4 hours. The reaction mixture was filtered and the organic phase concentrated under reduced pressure to afford the title compound as tan crystals, which were purified using HPLC-system II. (0.93 g, 94%; TLC *R_f_* 0.45, EtOAc/heptane, 1:2). ^1^H-NMR δ (ppm): 6.75 (s, 1H), 6.60 (s, 1H), 6.60 (d, *J* = 1.7 Hz, 1H), 6.18 (dt, *J* = 15.0 Hz, *J* = 1.5 Hz, 1H), 5.85 (s, 2H), 4.0 (m, 1H), 2.8 (t, 2H), 2.4 (t, 2H), 1.1 (m, 6H); MS: *m/z* 235.12 [M]^+^: HRMS (ESI+), M+Na^+^: 258.1106; calculated for C_13_H_17_NO_3_Na: 258.1106.
